# Supplementary material for: Hesperidin Anti-Osteoporosis by Regulating Estrogen Signaling Pathways
Source: Molecules. 2023 Oct 9;28(19):6987. doi: 10.3390/molecules28196987 (PMC10574669; doi:10.3390/molecules28196987)
Supplement: Supplementary file 1 [file molecules-28-06987-s001.zip › molecules-2623590-supplementary.pdf]

Table S1. Time Schedule for the Experiment on the Anti-Osteoporotic Effects of Hesperidin

Experiment Name	Experimental model	Groups	Incubation time	Culture conditions
Osteoblast proliferation	MC3T3-E1 cells	control group	3d	complete culture medium
		model group	3d	complete culture medium + dexamethasone (100 μmol/L)
		positive control group	3d	complete culture medium + dexamethasone + icariin (10 μg/mL)
		treatment group	3d	complete culture medium + dexamethasone + hesperidin (0.3125, 0.625, 1.25, 2.5, 5, 10, 20 μg/mL)
Alkaline phosphatase staining	MC3T3-E1 cells	control group	7d	complete culture medium
		model group	7d	osteogenic inducers (100 nmol/L dexamethasone, 50 μmol/L ascorbic acid, 10 mmol/L β-glycerophosphate)
		treatment group	7d	osteogenic inducers + hesperidin (1.25, 2.5, 5, 10 μg/mL)
Zebrafish bone protection	Zebrafish	control group	8d	culture medium (10% NaCl, 0.3% $CaCl_2$, 0.3% KCl, 0.79% $MgSO_4$)
		model group	8d	culture medium + prednisolone (20 mmol/L)
		positive control group	8d	culture medium + prednisolone + etidronate disodium (10 μg/mL)
		treatment group	8d	cultivation medium + prednisolone + hesperidin (1.25, 2.5, 5, 10 μg/mL)
Zebrafish oxidative stress	Zebrafish	control group	3d	culture medium
		model group	3d	culture medium +AAPH（20 mmol/L）
		treatment group	3d	cultivation medium + AAPH + hesperidin (1.25, 2.5, 5, 10 μg/mL)
